# Structural characterization of the circadian clock protein complex composed of KaiB and KaiC by inverse contrast-matching small-angle neutron scattering

**DOI:** 10.1038/srep35567

**Published:** 2016-10-18

**Authors:** Masaaki Sugiyama, Hirokazu Yagi, Kentaro Ishii, Lionel Porcar, Anne Martel, Katsuaki Oyama, Masanori Noda, Yasuhiro Yunoki, Reiko Murakami, Rintaro Inoue, Nobuhiro Sato, Yojiro Oba, Kazuki Terauchi, Susumu Uchiyama, Koichi Kato

**Affiliations:** 1 Research Reactor Institute, Kyoto University, Kumatori, Sennan-gun, Osaka 590-0494, Japan; 2 Graduate School of Pharmaceutical Sciences, Nagoya City University, 3-1 Tanabe-dori, Mizuho-ku, Nagoya 467-8603, Japan; 3 Okazaki Institute for Integrative Bioscience and 5-1 Higashiyama, Myodaiji, Okazaki, Aichi 444-8787, Japan; 4 Institut Laue-Langevin, 71, Avenue des Martyrs, Grenoble 38042, France; 5 Graduate School of Life Sciences, Ritsumeikan University, 1-1-1 Noji-higashi, Kusatsu, Shiga 525-8577, Japan; 6 Department of Biotechnology, Graduate School of Engineering, Osaka University, 2-1 Yamadaoka, Suita, Osaka 565-0871, Japan; 7 Institute for Molecular Sciences, National Institutes of Natural Sciences, 5-1 Higashiyama, Myodaiji, Okazaki, Aichi 444-8787, Japan

## Abstract

The molecular machinery of the cyanobacterial circadian clock consists of three proteins: KaiA, KaiB, and KaiC. Through interactions among the three Kai proteins, the phosphorylation states of KaiC generate circadian oscillations *in vitro* in the presence of ATP. Here, we characterized the complex formation between KaiB and KaiC using a phospho-mimicking mutant of KaiC, which had an aspartate substitution at the Ser431 phosphorylation site and exhibited optimal binding to KaiB. Mass-spectrometric titration data showed that the proteins formed a complex exclusively in a 6:6 stoichiometry, indicating that KaiB bound to the KaiC hexamer with strong positive cooperativity. The inverse contrast-matching technique of small-angle neutron scattering enabled selective observation of KaiB in complex with the KaiC mutant with partial deuteration. It revealed a disk-shaped arrangement of the KaiB subunits on the outer surface of the KaiC C1 ring, which also serves as the interaction site for SasA, a histidine kinase that operates as a clock-output protein in the regulation of circadian transcription. These data suggest that cooperatively binding KaiB competes with SasA with respect to interaction with KaiC, thereby promoting the synergistic release of this clock-output protein from the circadian oscillator complex.

Organisms on this planet exhibit circadian rhythms to adapt to daily alterations in the environment. Cyanobacteria are photoautotrophic organisms capable of oxygen-producing photosynthesis, similar to that observed in eukaryotic algae and plants, which exhibit circadian rhythms and have become one of the most useful model organisms for circadian biology^1^,^2^,^3^. The central oscillator that generates the circadian rhythm in the cyanobacterium *Synechococcus elongatus* PCC 7942 comprises only three proteins—KaiA, KaiB, and KaiC^4^. Through interactions among these proteins in the presence of ATP, KaiC undergoes phosphorylation and dephosphorylation cycles with the period of 24 h, which proceeds *in vitro* without daylight oscillation, indicating that the internal clock mechanism can be autonomous irrespective of transcriptional and translational feedback systems^5^,^6^,^7^.

KaiC forms a hexametric ring consisting of two rings—CI and CII^2^,^3^. Two specific residues positioned in the CII ring, S431 and T432, are phosphorylated and dephosphorylated in a 24-hour periodic manner^5^,^6^,^8^,^9^ as follows: KaiC-S/T → KaiC-S/pT → KaiC-pS/pT → KaiC-pS/T → KaiC-S/T, where S and T represent Ser431 and Thr432, respectively, and ‘p’ represents the phosphorylated residue. A series of phospho-mimicking KaiC mutants have generally been used for characterizing the functional roles of phosphorylation and the corresponding structural snapshots of the particular clock phase^10^,^11^,^12^. KaiA stimulates KaiC phosphorylation through the interaction of the A loops on the CII ring^13^, whereas KaiB accelerates the dephosphorylation process by interacting with the CI ring of phosphorylated KaiC^14^,^15^,^16^. During the latter process, KaiB undergoes a fold switch coupled with a transition from its homotetramerameric state to the KaiB–KaiC complex^10^. Formation of the KaiB–KaiC complex precludes the direct KaiA–KaiC interaction by promoting direct binding of KaiA to KaiB in the complex, thereby initiating a phase proceeding in the circadian cycle^10^,^16^. Furthermore, KaiB competes with SasA for binding to the KaiC hexamer. SasA is a histidine kinase that operates as a major clock-output protein that is released from the KaiABC clock oscillator during the regulation of circadian transcription^10^,^13^,^17^. Hence, the KaiB–KaiC interaction is a key event during oscillation of the cyanobacterial circadian protein system. Recent mass spectrometry (MS) analysis showed that KaiB can bind to phosphorylated KaiC at a stoichiometry of 6:6^18^. To further understand the circadian clock mechanisms of KaiB–KaC assembly, we collected detailed structural information on the protein complexes using inverse contrast-matching small-angle neutron scattering (iCM-SANS) in conjunction with native MS analyses.

## Results and Discussion

### Analysis of the interaction between KaiB and KaiC mutants

In this study, KaiC_AA_, KaiC_SE,_ KaiC_DT_, and KaiC_DE_ were used as phospho-mimicking mutants to examine which phosphorylation form of KaiC exhibits the highest binding affinity to KaiB. KaiC_DT_ and KaiC_SE_ are mono-phosphorylated KaiC mimics at Ser431 and Thr432, respectively, whereas KaiC_DE_ and KaiC_AA_ are hyperphosphorylated and hypophosphorylated mutants regarding these sites.

**Figure 1:**
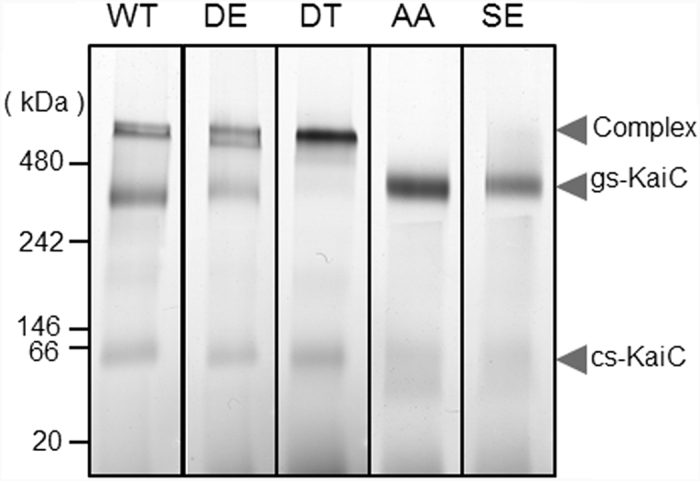
KaiC_DT_ predominantly binds to KaiB. KaiC_WT_, KaiC_DE_, KaiC_DT_, KaiC_AA_, and KaiC_SE_ were incubated with KaiB at 30 °C for 12 h and were subjected to BN-PAGE. The upper and lower bands with respect to the 480-kDa position corresponded to KaiB–KaiC complex and ground-state (gs)-KaiC, respectively. The 66-kDa protein bands corresponded to the competent state (cs)-KaiC. The gs-KaiC and cs-KaiC were donated in the previous work^21^. The full-length gels of BN-PAGE are presented in Supplemental Fig. S1.

Previous studies have indicated that KaiB is apt to bind phosphorylated KaiC mutants in which glutamate or aspartate residue occupies position 431 (e.g., KaiC_EE_, KaiC_DE_, KaiC_EA,_ or KaiC_DT_)^9^,^19^,^20^. Prior to our detailed structural analyses, we used Blue native-polyacrylamide gel electrophoresis (BN-PAGE) to investigate the binding abilities of KaiB to a series of KaiC phospho-mimic mutants, in which the KaiC hexamer formed two conformational states: ground-state (gs)-KaiC, which was stable, and competent-state (cs)-KaiC (Fig. 1), which was labile and degraded into monomers upon binding of Coomassie Brilliant Blue)^21^. KaiB forms stable complexes with wild-type KaiC (KaiC_WT_) as well as KaiC_DE_ and KaiC_DT_ mutants. KaiC_DT_ mimicked an optimally phosphorylated form of KaiC in terms of KaiB binding in comparison with KaiC_WT_ and the other KaiC mutants, KaiC_DE_, KaiC_AA_, and KaiC_SE_ (Fig. 1). Therefore, the interaction between KaiB and KaiC_DT_ was further characterized.

### Oligomeric state of the KaiB–KaiC complex

**Figure 2:**
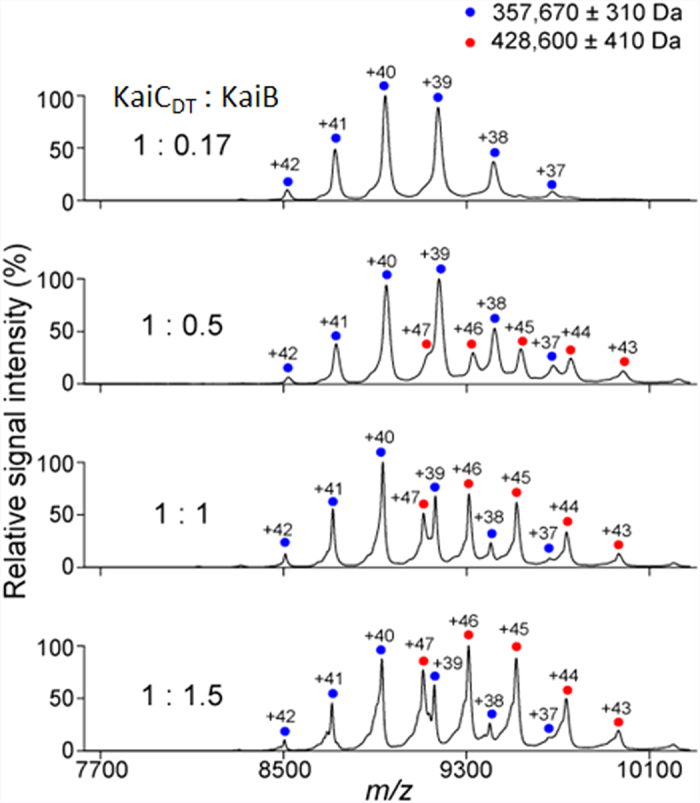
Characterization of the KaiB–KaiC_DT_ complex. Mass spectra of mixtures of KaiC_DT_ and KaiB at 1:0.17, 1:0.5, 1:1, and 1:1.5 molar ratios (KaiC_DT_ to KaiB). Blue and red circles show the ion series of the KaiC_DT_ homo-hexamer and the 6:6 hetero-dodecamer complexes of KaiC_DT_ and KaiB, respectively.

To characterize the oligomeric state of the KaiB–KaiC complex in solution, we performed native MS and sedimentation velocity analytical ultracentrifugation (SV-AUC) analyses. Our native MS data indicated that upon titration with KaiB, the KaiC_DT_ hexamer formed a uniform complex with a molecular mass of 428,600 ± 410 Da, which corresponded to a 6:6 stoichiometry (Fig. 2 and Supplemental Fig. S2), consistent with previous native MS data obtained using hyperphosphorylated KaiC_WT_^18^. Our titration results underscore the fact that the complex formed between KaiB and KaiC_DT_ only existed in a 6:6 stoichiometry, indicating that the six KaiB protomers bind to the KaiC hexamer with strong positive cooperativity. The SV-AUC data confirmed that KaiC_DT_ forms a homogeneous hexamer and that KaiB and KaiC_DT_ form a 6:6 complex with a sedimentation coefficient of 13.7 S (Supplemental Fig. S3).

### Spatial arrangement of the KaiB subunits in the KaiB–KaiC complex

We analyzed the spatial arrangement of the KaiB subunits in the 6:6 KaiB–KaiC_DT_ complex in an aqueous solution by small-angle scattering. In this approach, the scattering intensity arising from each domain is proportional to the square of its molecular mass. This means that in conventional small-angle scattering such as small-angle X-ray scattering, scattering from the much larger KaiC (with a molecular mass of 65 kDa) dominates that of KaiB (with a molecular mass of 14 kDa). Therefore, it is difficult to collect structural information on KaiB from the scattering profile of the complex. Our strategy for overcoming this difficulty was to weaken or erase scattering from the KaiC subunits in the complex using the iCM-SANS method^22^. In neutron scattering, an isotope effect is most remarkable between hydrogen and deuterium: the scattering lengths of hydrogen and deuterium are −3.74 fm and +6.67 fm, respectively. This enables control of the scattering length densities of the solvent and the properly deuterated protein components in the complex^23^,^24^, facilitating elimination of the scattering originating from those protein components^22^.

**Figure 3:**
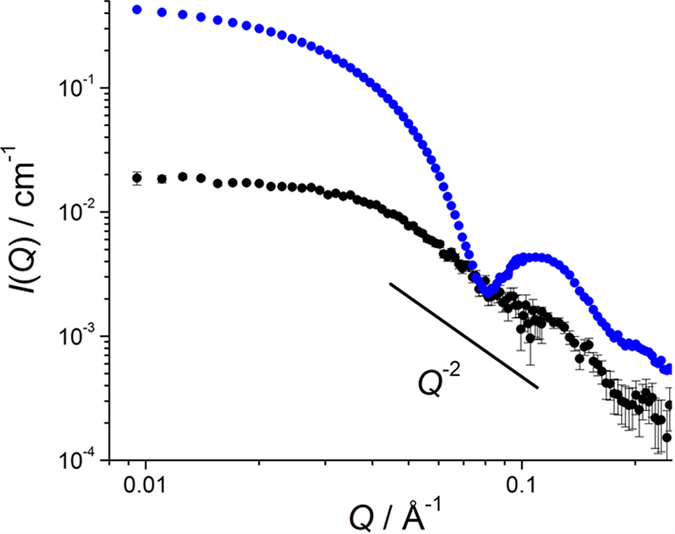
SANS profiles of 72d-KaiC_DT_+h-KaiB (black) and h-KaiC_DT_+h-KaiB (blue) complexes in 97% D_2_O.

In this study, we prepared the 6:6 KaiB–KaiC_DT_ complex consisting of the partially deuterated KaiC_DT_ subunits and the nondeuterated KaiB (h-KaiB) subunits. The solvent D_2_O ratio at the contrast matching point was determined to eliminate scattering originating from the partially deuterated KaiC_DT_ subunits. The observed SANS intensity at 0.029 Å^−1^, *I*_O_(0.029), of the partially deuterated KaiC_DT_ protein alone dissolved in 0% D_2_O at a concentration of 1 mg/mL, was found to be 0.575 ± 0.003 cm^−1^ (Supplemental Fig. S4). In addition, *I*_C_(0.029) was calculated as a function of the protein deuteration ratio based on the amino-acid sequence of KaiC_DT_. From the square roots of *I*_O_(0.029) and *I*_C_(0.029), the deuteration ratio of the partially deuterated KaiC_DT_ preparation was found to be 72.2% (Supplemental Fig. S5). Hereafter, this KaiC_DT_ preparation will be referred to as 72d-KaiC_DT_. In 97% D_2_O solvent, 72d-KaiC_DT_ was virtually invisible in terms of scattering because the scattering length density of KaiC_DT_ was matched to that of the solvent (Supplemental Figs S4 and S6). As a result, we could clearly exclusively observe the neutron scattering of KaiB subunits in the complex. Figure 3 compares the SANS profiles of 72d-KaiC_DT_+h-KaiB and h-KaiC_DT_+h-KaiB complexes in 97% D_2_O. The scattering of 72d-KaiC_DT_+h-KaiB complex showed marked differences from that of the h-KaiC_DT_+h-KaiB complex in which both KaiB and KaiC fully contributed to scattering. In addition, the scattering profile of the 72d-KaiC_DT_+h-KaiB complex approximately followed the *Q*^−2^ power law indicating that the six KaiB subunits were arranged into a disk-like shape^25^.

**Figure 4:**
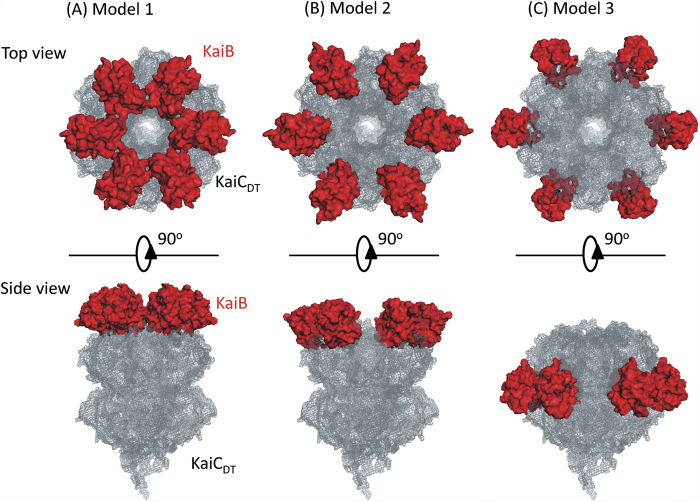
Structural models of the KaiB–KaiC_DT_ complex.

With the assumption of six-fold symmetry of arrangement of the six KaiB subunits along with their KaiC-binding surfaces consistent with the previously reported deuterium exchange mass spectrometric data^10^, we examined the following three distinct structural models of the KaiB–KaiC_DT_ complex: Model 1: The KaiB subunits form a hexameric ring on the top of the KaiC C1-ring. The distance between the center of mass of each KaiB component and the six-fold axis of KaiC_DT_ is set to be 33 Å, which is the minimum distance with avoidance of steric hindrance between the KaiB subunits (Fig. 4A). Model 2: The KaiB subunits are located on the edge of the KaiC C1-ring. The distance between the center of mass of KaiB and the six-fold axis of KaiC_DT_ is set to be 45 Å, which is the maximum distance with direct interactions between the KaiB and KaiC_DT_ components (Fig. 4B). Model 3: The KaiB subunits are located on the side of the KaiC C1-ring with 60 Å of the distance between center of mass of KaiB and the six-fold axis of KaiC_DT_ (Fig. 4C).

**Figure 5:**
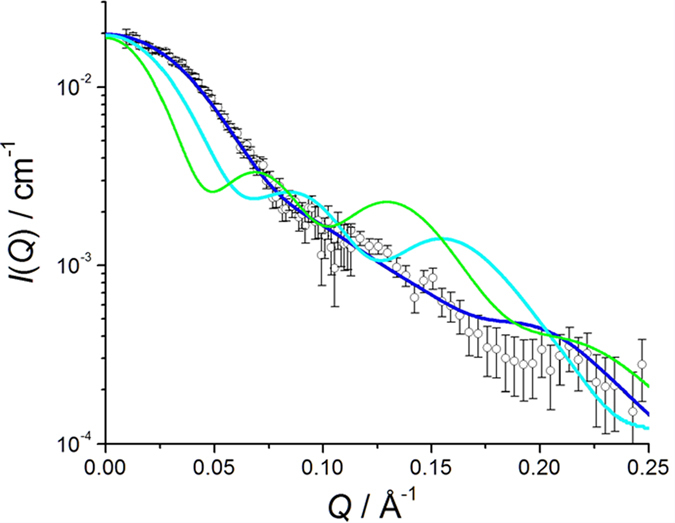
SANS profiles calculated from Model 1 (blue), Model 2 (cyan), and Model 3 (green) along with the experimentally obtained profile (open circle).

The scattering profiles with 72d-KaiC and h-KaiB in 97% D_2_O were calculated for each model and compared with the experimental data (Fig. 5). The results clearly showed that Model 1 illustrated the best structural arrangement of the KaiB subunits in the complex. Furthermore, the experimentally determined gyration radius, 33.6 Å, was consistent with that computed from Model 1, 33.2 Å, in comparison with those from Model 2 (44.2 Å) and Model 3 (59.4 Å). The hexameric KaiB ring forming on the KaiC hexamer was consistent with C6-symmetrized particle-image-based cryoEM structure^26^. In this interaction mode, the hexameric KaiB subunits cover the top surface of the KaiC C1 ring including the interaction site of SasA^10^. Therefore, it is conceivable that the cooperative binding of KaiB as competitive inhibitor against SasA promotes synergistic release of this clock-output protein from the KaiC hexamer.

In summary, we obtained structural information on the KaiB–KaiC complexes using iCM-SANS in conjunction with native MS analyses, providing insights into the working mechanisms of the circadian clock comprised of Kai proteins.

## Methods

### Protein expression and purification

KaiA, KaiB, and KaiC originating from *S. elongatus* PCC 7942 were expressed in *Escherichia coli* as Strep-tagged recombinant proteins and purified as previously described^21^. The expression plasmids of the KaiC mutants (KaiC_DE_, KaiC_DT_, KaiC_AA_, and KaiC_SE_) were also constructed according to a previous study^21^. KaiC_DE_ is a mutant of KaiC with aspartate and glutamate residues at positions 431 and 432, respectively, that mimics hyperphosphorylated KaiC, whereas KaiC_AA_ is a mutant with alanine residues at these positions that mimics hypophosphorylated KaiC. For preparation of the deuterated proteins, the bacterial cells were grown in M9 minimal media containing glucose as a mixture with varying ratios of isotopically natural and fully deuterated glucose (1,2,3,4,5,6,6-D_7_, 98%, Cambridge Isotope Laboratories, Inc.), along with varying ratios of H_2_O and D_2_O as previously described^23^,^24^.

### BN-PAGE

BN-PAGE was performed using a NativePAGE^TM^ Novex Bis-Tris Gel System (Invitrogen) according to the manufacturer’s protocols with some modifications as previously described^21^.

### Analytical ultracentrifugation

The sedimentation velocity method was used to characterize KaiB, KaiC_DT_, and their complex. Concentrations of KaiB, KaiC_DT_, and their complex were 40 μM, 30 μM, and 60 μM, respectively, in 20 mM Tris-HCl (pH 8.0), 150 mM NaCl, 1 mM ATP, 5 mM MgCl_2_, 1 mM DTT, and 0.5 mM EDTA. The samples were placed in analytical cells with double-sector centerpieces with sapphire windows. The experiments were performed using an Optima XL-I analytical ultracentrifuge (Beckman-Coulter) at 20 °C and an angular velocity of 50,000 rpm for KaiB, 30,000 rpm for KaiC_DT_ and 20,000 rpm for their complex. Data were recorded with Rayleigh interference optical system, followed by the analysis with a c(*s*) distribution of the Lamm equation solutions calculated by the Sedfit v 14.4^27^. The partial specific volume of the KaiB, 0.7556 ml/g, KaiC_DT_, 0.7361 ml/g, and their complex, 0.7458 ml/g were calculated from their amino acid compositions using the program SEDNTERP ver.1.09, buffer density (1.00540 g/cm^3^), and buffer viscosity (1.0245 cP) were estimated using the program SEDNTERP ver.1.09.

### MS under non-denaturing conditions

The purified KaiC_DT_ and KaiB proteins (20 μM and 3.3, 10, 20, and 30 μM monomer, respectively) were mixed, incubated at 30 °C for 4 h, and buffer-exchanged into 150 mM ammonium acetate, pH 7.5, by passing the proteins through a Bio-Spin 6 column (Bio-Rad). The buffer-exchanged KaiCDT–KaiB complexes were immediately analyzed by nanoflow electrospray ionization MS using gold-coated glass capillaries made in house (approximately 2–5 μL sample loaded per analysis). Spectra were recorded on a SYNAPT G2-S*i* HDMS mass spectrometer (Waters, Milford, MA, USA) in positive ionization mode at 1.33 kV with a 150 V sampling cone voltage and source offset voltage, 0 V trap and transfer collision energy, and 5 mL/min trap gas flow. The spectra were calibrated using 1 mg/mL cesium iodide and analyzed using MassLynx software (Waters).

### SANS measurements

SANS experiments were performed using the D22 instrument installed at the Institut Laue-Langevin (ILL), Grenoble, France. The SANS intensities were observed with 6.0 Å neutrons and two sample-to-detector distances of 5.6 m and 2.0 m: the covered *q*-ranges are 0.0095 to 0.25 Å^−1^. The temperature was maintained at 20 °C in the irradiation. The observed SANS intensity was corrected for background, empty cell and buffer scatterings, and transmission factors and subsequently converted to the absolute scale by GRASP software using incident beam flux (http://www.ill.eu/instruments-support/instruments-groups/groups/lss/grasp/home/). For SANS measurements, 1–2 mg/mL KaiC was dissolved in buffer containing 50 mM NaH_2_PO_4_ (pH 7.8), 150 mM NaCl, 1 mM ATP, 5 mM MgCl_2_, 1 mM DTT, 0.5 mM EDTA, 50 mM L-arginine, 50 mM L-glutamic acid, and various concentration of D_2_O (ISOTECH) in presence and absence of KaiB. All SANS samples are listed in Table S1.

### 3D structure modeling

For simulation of the SANS profile of KaiB, the 3D-structural model of a fold switch KaiB with strept tag was developed on the basis of SWISS-MODEL^28^,^29^,^30^. Furthermore, 3D models of the KaiB–KaiC complex were built by arranging six KaiB subunits on hexametric KaiC ring with a pseudo-sixfold symmetry. In all three models, the interaction surface of KaiB is facing KaiC_DT_ (Supplemental Fig. S7).

## Additional Information

**How to cite this article**: Sugiyama, M. *et al*. Structural characterization of the circadian clock protein complex composed of KaiB and KaiC by inverse contrast-matching small-angle neutron scattering. *Sci. Rep.* **6**, 35567; doi: 10.1038/srep35567 (2016).

## Supplementary Material

Supplementary Information
