# Supplementary material for: Structural characterization of the circadian clock protein complex composed of KaiB and KaiC by inverse contrast-matching small-angle neutron scattering
Source: Sci Rep. 2016 Oct 18;6:35567. doi: 10.1038/srep35567 (PMC5067715; doi:10.1038/srep35567)
Supplement: Supplementary Information [file srep35567-s1.pdf]

Supplemental information

Structural characterization of the circadian clock protein complex composed of KaiB and KaiC by inverse contrast-matching small-angle neutron scattering

Masaaki Sugiyama[a,1,2], Hirokazu Yagi[b,1], Kentaro Ishii[c], Lionel Porcar[e], Anne Martel[e], Katsuaki Oyama[f], Masanori Noda[g], Yasuhiro Yunoki[b], Reiko Murakami[b], Rintaro Inoue[a], Nobuhiro Sato[a], Yojiro Oba[a], Kazuki Terauchi[f], Susumu Uchiyama[c,g], and Koichi Kato[b,c,d,2]

[a]Research Reactor Institute, Kyoto University, Kumatori, Sennan-gun, Osaka 590-0494, Japan

[b]Graduate School of Pharmaceutical Sciences, Nagoya City University, 3-1 Tanabe-dori, Mizuho-ku, Nagoya 467-8603, Japan

[c]Okazaki Institute for Integrative Bioscience and [d] Institute for Molecular Sciences, National Institutes of Natural Sciences, 5-1 Higashiyama, Myodaiji, Okazaki, Aichi 444-8787, Japan

[e]Institut Laue-Langevin, 6 Rue Jules Horowitz, Grenoble 38042, France

[f]Department of Life Sciences, Ritsumeikan University, 1-1-1 Noji-higashi, Kusatsu, Shiga 525-8577, Japan

[g]Department of Biotechnology, Graduate School of Engineering, Osaka University, 2-1 Yamadaoka, Suita, Osaka 565-0871, Japan

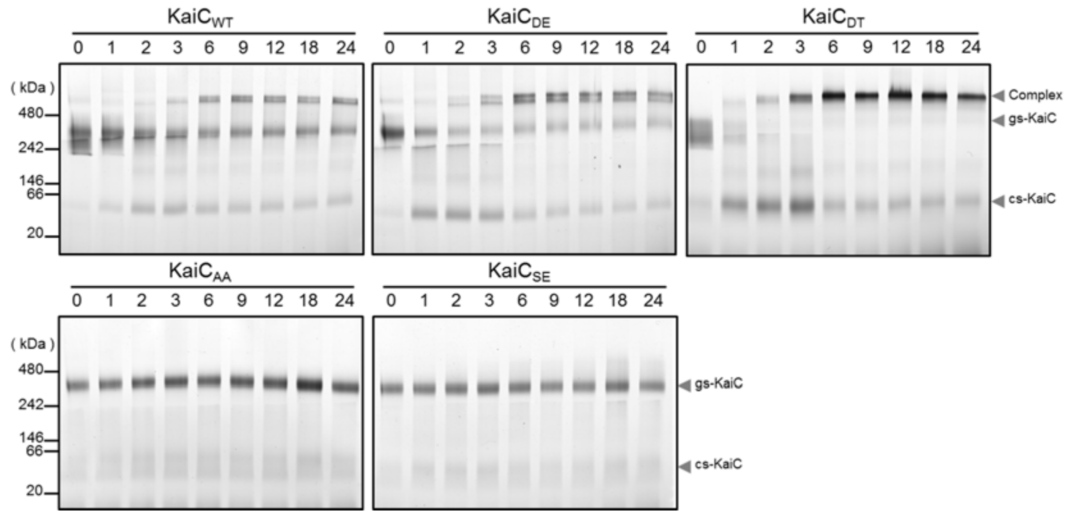

Supplemental Fig. S1. Full-length BN-PAGE images of Fig.1. KaiC$_{WT}$, KaiC$_{DE}$, KaiC$_{DT}$, KaiC$_{AA}$, and KaiC$_{SE}$ were incubated with KaiB at 30ºC and were subjected to BN-PAGE at individual time points. The 12hr-lanes of individual BN-PAGE images are highlighted in Fig. 1.

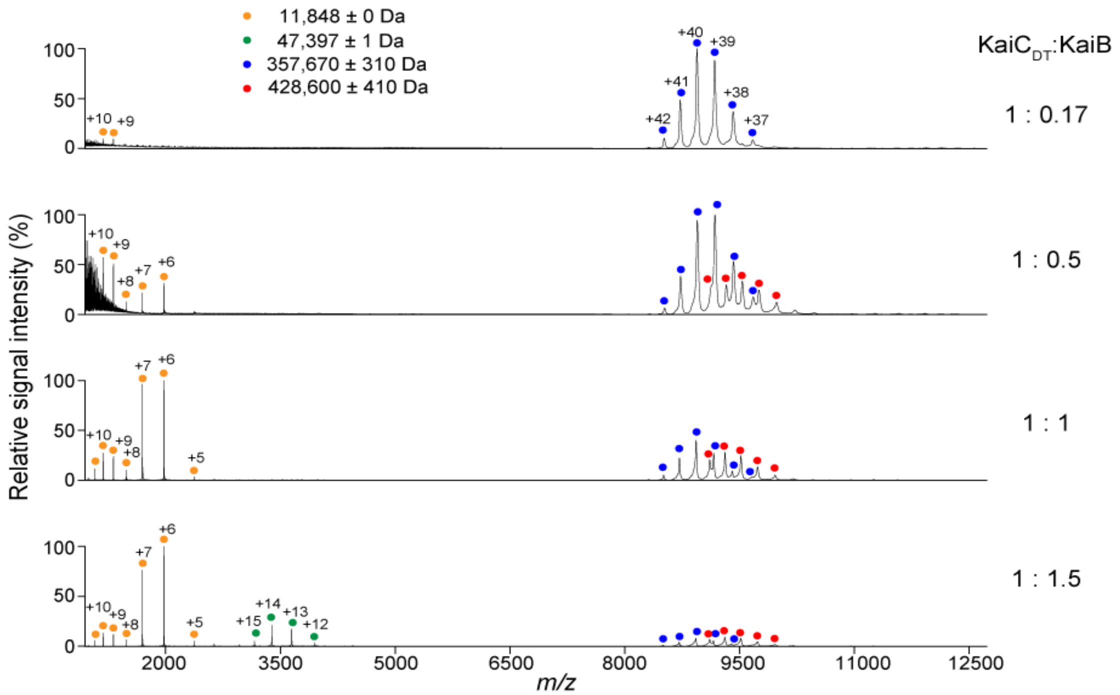

Supplemental Fig. S2. Mass spectra of mixtures of KaiC$_{DT}$ and KaiB at 1:0.17, 1:0.5, 1:1, and 1:1.5 molar ratios (KaiC$_{DT}$ to KaiB). The mass spectra in the range from *m/z* 1,000 to *m/z* 12,700 in Figure 2 are shown. Orange, green, blue, and red circles show the ion series of the monomeric KaiB, the homo-tetrameric KaiB, the homo-hekameric KaiC$_{DT}$, and the 6:6 hetero-dodecamer complexes of KaiC$_{DT}$ and KaiB, respectively.

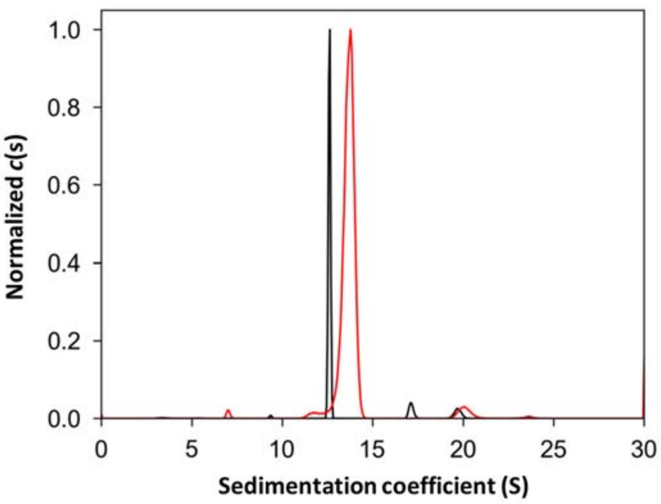

Supplemental Fig. S3. Distribution of sedimentation coefficients of 60 μM $KaiC_{DT}$ (black) and 60 μM KaiB- $KaiC_{DT}$ complex (red). The 13.7 S peak corresponds to the complex at a 6:6 stoichiometry.

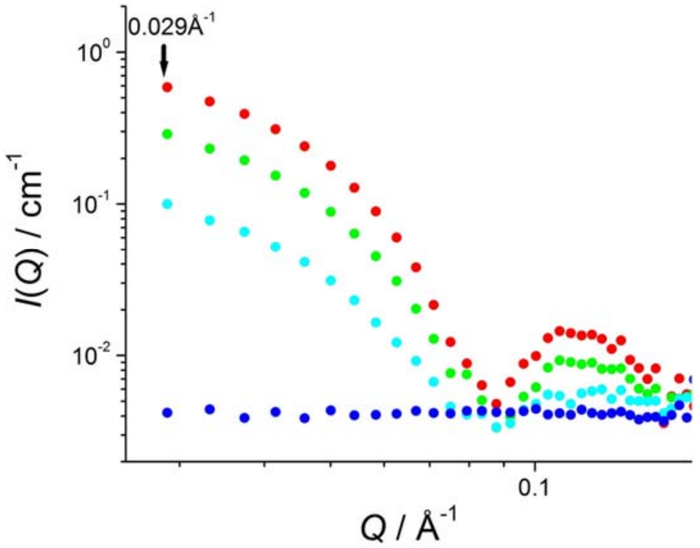

Supplemental Fig. S4. SANS profiles of the partially deuterated $KaiC_{DT}$ preparation in 0% (red), 30% (green), 60% (cyan), and 97% (blue) D_2O solvents. The profiles were measured with 6.0 Å neutron and the sample-to-detector distance of 2.0 m in 300 s by D22 of ILL.

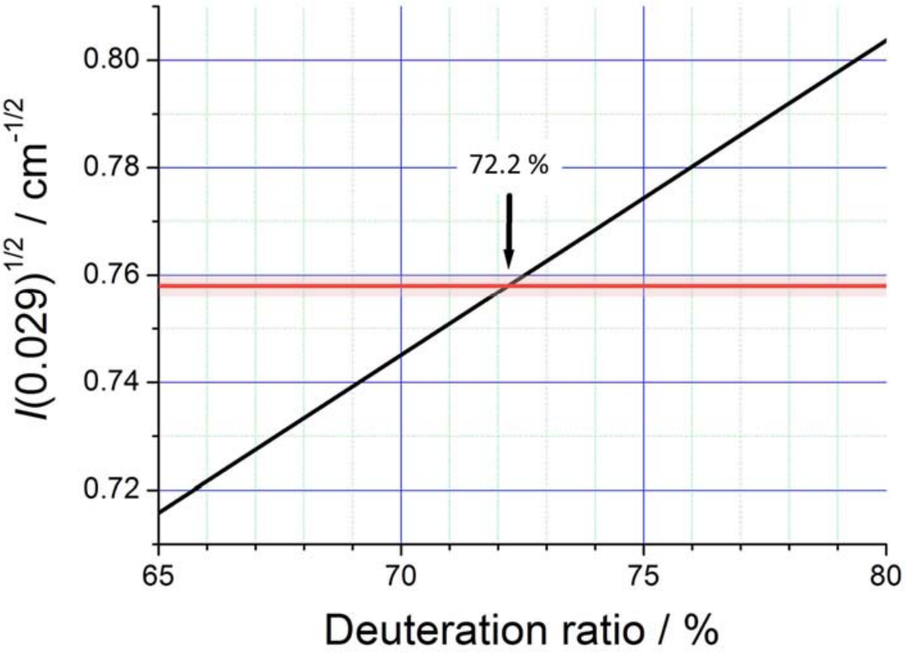

Supplemental Fig. S5. Square root of SANS intensity at 0.029 Å^{-1}, $I(0.029)^{1/2}$, in 0% D_2O (H_2O), of partially deuterated KaiC$_{DT}$. The black line indicates the calculated $I_C(0.029)^{1/2}$ as a function of deuteration ratio, while the red line and the orange zone show the observed value and the errors of $I_O(0.029)^{1/2}$ of the partially deuterated KaiC$_{DT}$ preparation, respectively. The cross point of black and red lines indicates that the deuteration ratio of the sample was 72.2 ± 0.3%.

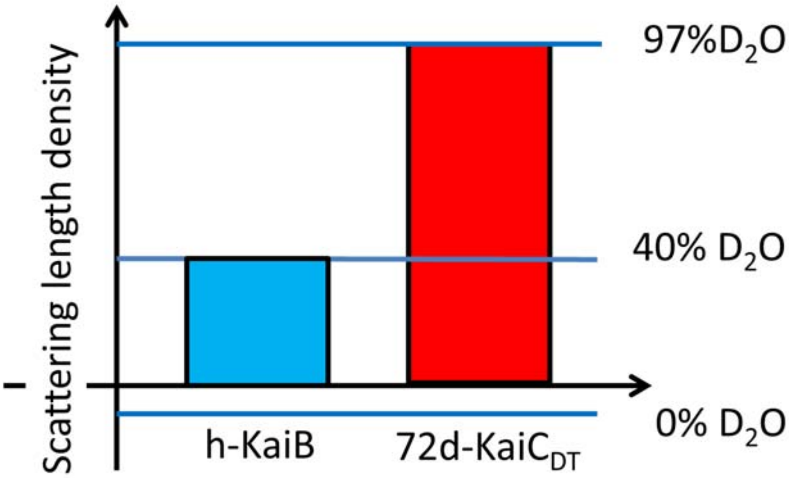

Supplemental Fig. S6. Map of scattering length density. Blue and red bars show the scattering length densities of hydrogenated KaiB (h-KaiB) and 72% deuterated KaiC$_{DT}$ (72d-KaiC$_{DT}$). The scattering length densities of 0%, 40%, and 97% D_2O express with blue cross bars. Because a scattering intensity of substance in a solution is proportional to the square of the difference in scattering length density between solute and solvent, namely "contrast," the scattering of 72d-KaiC$_{DT}$ can be almost ignored compared from that of h-KaiB in 97% D_2O solvent: yellow arrow shows the contrast of h-KaiB in 97% D_2O solvent.

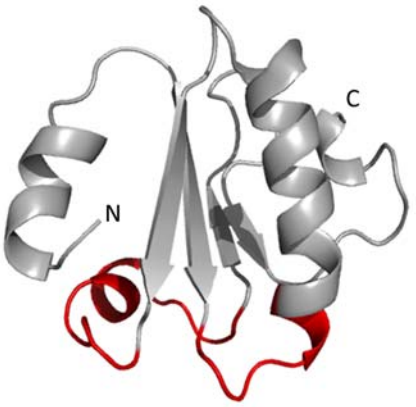

Supplemental Fig. S7. The KaiC-binding site in the structural model of the fold switch KaiB used for calculation of SANS profiles. The KaiC-binding site (red) was mapped on the basis of the previously reported deuterium-exchange MS data[1].

Supplemental Table S1: Samples used for SANS experiments.

<table>
<tr><th>Protein</th><th>Protein deuteration ratio</th><th>Solvent D_2O ratio</th><th>Figure of SANS profile</th></tr>
<tr><td>$KaiC_{DT}$</td><td>72%</td><td>0%</td><td>Fig. S3</td></tr>
<tr><td>$KaiC_{DT}$</td><td>72%</td><td>30%</td><td>Fig.S3</td></tr>
<tr><td>$KaiC_{DT}$</td><td>72%</td><td>60%</td><td>Fig.S3</td></tr>
<tr><td>$KaiC_{DT}$</td><td>72%</td><td>97%</td><td>Fig.S3</td></tr>
<tr><td rowspan="2">h-KaiB+
h-$KaiC_{DT}$</td><td>0%</td><td rowspan="2">97%</td><td rowspan="2">Fig.3</td></tr>
<tr><td>0%</td></tr>
<tr><td rowspan="2">h-KaiB+
d-$KaiC_{DT}$</td><td>0%</td><td rowspan="2">97%</td><td rowspan="2">Figs. 3 and 5</td></tr>
<tr><td>72%</td></tr>
</table>

Reference

1. Chang, Y.G. et al. Circadian rhythms. A protein fold switch joins the circadian oscillator to clock output in cyanobacteria. *Science* **349**, 324-8 (2015).
